# Functional imaging of hepatocellular carcinoma using diffusion-weighted MRI and ^18^F-FDG PET/CT in patients on waiting-list for liver transplantation

**DOI:** 10.1186/s40644-016-0062-8

**Published:** 2016-02-16

**Authors:** Samia Boussouar, Emmanuel Itti, Shih-Jui Lin, Thomas Decaens, Eva Evangelista, Melanie Chiaradia, Julia Chalaye, Laurence Baranes, Julien Calderaro, Alexis Laurent, Frederic Pigneur, Christophe Duvoux, Daniel Azoulay, Charlotte Costentin, Alain Rahmouni, Alain Luciani

**Affiliations:** AP-HP, Hôpitaux Universitaires Henri Mondor, Imagerie Médicale, Créteil, F-94010 France; Université Paris Est Créteil, Faculte de Médecine, Créteil, F-94010 France; AP-HP, Hôpitaux Universitaires Henri Mondor, Médecine Nucléaire, Créteil, F-94010 France; INSERM Unité U 955, GRC Amyloid Research Institute, Créteil, F-94010 France; Biomedical Informatics, Stanford University, Stanford, CA 94305 USA; AP-HP, Hôpitaux Universitaires Henri Mondor, Hépatologie, Créteil, F-94010 France; AP-HP, Hôpitaux Universitaires Henri Mondor, Pathologie, Créteil, F-94010 France; INSERM Unité U 955, Equipe 18, Créteil, F-94010 France; AP-HP, Hôpitaux Universitaires Henri Mondor, Chirurgie hépatique, Créteil, F-94010 France; Service de Médecine Nucléaire, CHU Henri Mondor, 51 Avenue du Marechal de Lattre de Tassigny, 94010, Créteil, Cedex France

**Keywords:** Hepatocellular carcinoma, Positron emission tomography, Diffusion-weighed imaging

## Abstract

**Background:**

To compare the apparent diffusion coefficient (ADC) on diffusion-weighted imaging (DWI) with the standardized uptake values (SUV) measured by^18^F-FDG-PET/CT in naïve hepatocellular carcinoma (HCC) nodules, and to determine whether these markers are associated with tumours at high-risk of aggressiveness.

**Methods:**

From 2007 to 2010, all patients with HCC on the waiting list for liver transplantation and who underwent both FDG-PET/CT and 1.5-T DWI-MRI (b values: 0, 200, 400, and 800 s/mm^2^) were included in this institutional review board-approved retrospective study. Tumour size, tumour ADC, tumour-to-liver ADC ratio (ADC_T/L_), maximal tumour SUV and tumour-to-liver SUV ratio (SUV_T/L_) were measured and compared to serum alpha-fetoprotein (AFP) levels, tumour size and differentiation grade on explanted specimens.

**Results:**

A total of 37 HCC nodules in 28 patients were available for correlation between MRI and PET/CT, 7 of which (in 7 patients) showed a SUV_T/L_ > 1.15. We did not find any correlation between tumour ADC or ADC_T/L_ and tumour SUV or SUV_T/L_. To note, SUV_T/L_ was positively correlated with AFP levels (*R* = 0.95, P ≤ 0.0001), while ADC_T/L_ was not (*P* = 0.73). Twenty-four patients (with 32 nodules) underwent liver transplantation. In this subgroup, an increased SUV_T/L_ ratio was associated with larger tumours (average size, 32 ± 14 mm; range, 18–60 mm; *P* < 0.0001) and with poor differentiation on pathology (grades 3 and 4; *P* = 0.04), while ADC_T/L_ was neither associated with tumour size or differentiation grade.

**Conclusions:**

ADC and SUV measures in HCC nodules are not correlated. SUV_T/L_ ratio correlates with AFP levels, tumour size and poor differentiation, and should probably be integrated as a co-variable in a predictive outcome model of patients on the waiting-list for liver transplantation.

## Background

While the prognosis of hepatocellular carcinoma (HCC) without treatment is sombre, with 5-year life expectancy below 7 %, optimal outcome is obtained in patients with low tumour burden, eligible for liver transplantation (LT) [1], especially within the Milan criteria [2]. Several studies have reported that patients transplanted beyond the Milan criteria can achieve long-term recurrence-free survival: pathological features of HCC tumours appear to play an important role as prognostic markers of disease-free survival; increased alpha-fetoprotein (AFP) levels, micro vascular invasion (MVI), differentiation grade, and satellite nodules all appear to be associated with impaired outcome following LT [3–9]. As a result, the sole assessment of the number and size of HCC lesions in LT candidates is unable to precisely address the risk of cancer recurrence after LT. As some pathological prognostic markers such as MVI cannot be assessed on biopsy specimens, growing interest has raised for the evaluation of HCC using functional imaging as an adjunct to biology and conventional imaging for optimal tumour grading.

Several studies have reported that pre-LT PET/CT with [^18^F] 2- deoxy-2-fluoro-D-glucose (FDG) may play a role in predicting tumour recurrence [10–15] in high-grade HCC tumours, which have a reduced glucose-6-phosphatase activity, resulting in progressive accumulation of the tracer within tumour cells as compared to adjacent liver. Hence, FDG standardized uptake value (SUV) in HCC, as compared to adjacent normal liver (tumour-to-liver ratio), reflects tumour aggressiveness, including histological tumour grade and AFP levels, which in turn correlate with outcome. For Lee et al., a longer disease-free survival was observed after LT in patients with a tumour-to-liver ratio < 1.15, i.e., with less than 15 % higher FDG uptake above normal liver background [16]. These data were later confirmed by Kornberg et al., demonstrating the pejorative predictive value of FDG-positive HCC [17].

Diffusion-weighted imaging (DWI) provides information on the random Brownian motion of water molecules within tissues, which reflects their cellularity. Some authors have reported that high-grade HCC tended to show decreased apparent diffusion coefficient (ADC) values, as compared to well-differentiated tumours [18], although this remains to-date debated [19]. Hence, both SUV and ADC could be considered as biomarkers of HCC tumours at risk of recurrence following LT, and could thereafter be tested as diagnostic and prognostic tools for HCC management. To the best of our knowledge, however, there have been no reports correlating both markers in HCC. The aim of our study was to measure and compare the ADC values on DWI with SUVs measured by FDG-PET/CT in naïve HCC patients on waiting-list for LT, and to determine whether these markers can be associated with tumours at high risk of aggressiveness using the pathology of explanted specimens as the reference standard.

## Methods

### Patients

The local patient database was searched retrospectively to identify all consecutive patients with HCC referred to our institution for LT between 2007 and 2010, who underwent both liver MRI and FDG-PET/CT prior to LT as part of their work-up. During this period, 78 patients met the inclusion criteria. We excluded patients in whom DWI sequences were not performed (*n* = 15), patients bearing liver lesions all < 10 mm on MRI (*n* = 13) and patients who underwent percutaneous tumour ablation or chemoembolization prior to either PET/CT or liver MRI (*n* = 22). As a result, 28 patients were eligible, including 23 men and 5 women, with a median age of 59 years (range, 46 to 69 years); 9 patients were Child-Pugh A, 15 Child-Pugh B, and 4 Child-Pugh C; the underlying liver disease in the study population is shown in Table 1. Based on pre-LT imaging data, 67.8 % (19/28) patients fulfilled the Milan criteria (2), and 82.1 % (23/28) fulfilled the UCSF criteria [20]. All were included in clinical trials, which were active at this time, and signed informed written consent, in accordance with our Institutional Review Board (Comité de Protection de Personnes, Ile-de-France IX).

**Table 1:** Patient population features

Patients (n)	28
Age (years) [Mean (±SD)]	59 ± 6
Sex ratio (Male/Female)	23/5
Underlying liver disease (n):	
Alcohol	10
Hepatitis B	5
Hepatitis C	4
NASH	2
NASH + alcohol	2
Hepatitis C + alcohol	2
Hepatitis C + alcohol + hemochromatosis	1
Hemochromatosis + hepatitis C	1
NASH + hemochromatosis	1

*NASH* Non Alcoholic Steato-Hepatitis

Fifteen patients underwent neo-adjuvant treatments before LT, all of which were performed after both the liver MRI and PET/CT: 13 patients underwent lipiodol-doxorubicin based chemoembolization, either alone (*n* = 8) or combined with radiofrequency ablation (*n* = 3) and surgery (*n* = 2); 2 patients underwent radiofrequency ablation prior to LT. MRI and PET/CT comparisons were performed on treatment-naive HCC patients.

### Magnetic resonance imaging

All MRI images were acquired on a 1.5-T MR imaging system (Avanto®, Siemens Healthcare, Erlangen, Germany) with Super Quantum gradients (maximum gradient amplitude 40 mT/m, maximal gradient slope 200 mT/m), and an 18-channel total imaging matrix system. The liver MRI protocol included transverse gradient recalled echo (GRE) in and out-of-phase T1 sequences (TR/TE/α: 119 ms/2.4–4.8 ms/70°; slice thickness 5 mm), and breath-hold fat suppressed transverse turbo spin echo (TSE) T2 sequences (TR/TE/α: 2400 ms/82 ms/150°; slice thickness 5 mm; echo train length = 23). The DWI sequence was based on standard single-shot diffusion-weighted spin echo planar imaging with 4 b-values (0, 200, 400 and 800 s/mm^2^), and was acquired prior to Gd-chelates injection. The DWI sequence was acquired with respiratory gating (Prospective Acquisition Correction [PACE] system, average TR = 1500 ms, TE = 78 ms; slice thickness 5 mm; matrix 148x112; FOV 300x250 mm; Nex = 3; BW = 1342 Hz/pixel; generalized autocalibrating partially parallel acquisition factor 2). A dynamic breath-hold 3D volumetric interpolated breath-hold examination (VIBE) T1WI (TR/TE/α: 3.17 ms/1.33 ms/20°; slice thickness 3 mm; mean acquisition time of 19 ± 3 s) was repeated before and 4 times after injection of a total of 0.2 mL/kg gadoteric acid (Dotarem®, Guerbet, Aulnay, France). Each 3D VIBE was acquired at a 20 s interval.

All MR images were analyzed by 2 observers (SB, ALu), blinded to PET/CT results. For each patient, the number of lesions fulfilling the European Association for the Study of Liver Disease and American Association for the Study of Liver Disease (EASL-AASLD) [21] diagnostic criteria on MRI was determined, thereafter defining whether each patient fulfilled the Milan and UCSF criteria [2, 20]. Analyses were performed on a commercially available Siemens Workstation (Syngo MMWP, Siemens Healthcare, Forchheim, Germany). In addition, for each HCC nodule, the following parameters were measured: size, location according to Couinaud liver segmentation, T1 signal intensity (SI) relative to muscle, T2SI relative to spleen. ADC maps were obtained using a mono-exponential fit of the signal decrease on the 4 b values on a voxel-by-voxel basis. A region of interest (ROI) encompassing the entire lesion was placed on the b0 image, and then, propagated to the ADC map to provide the mean tumour ADC (ADC_T_). The ADC of the normal liver parenchyma (ADC_L_) was also measured on the right posterior segment of the liver, using a 20 mm ROI positioned away from tumours, vessels, and bile ducts. The tumour-to-liver ratio (ADC_T/L_) was computed for each HCC lesion.

### PET/CT imaging

The median delay between PET/CT and MRI was 3 days (range, −181 to 245 days). All images were acquired using an integrated PET/CT scanner (Gemini GXL16, Philips, Da Best, The Netherlands). After fasting for at least 6 h, patients were injected with an average 5.2 MBq/kg FDG, and images were acquired 1 h later. A low-dose CT scan was obtained for attenuation correction and localization purposes (100 kV, 80 mAs), followed by an emission scan from the top of the skull to mid-thigh with 9–11 bed shifts of 2 min each. Images were reconstructed using a line-of-response row action maximum likelihood algorithm (LOR-RAMLA) with matrix size of 144x144, and corrections for attenuation, scatter and random coincidences. CT was obtained without contrast enhancement.

PET, CT, and fused PET/CT images were analyzed on a proprietary workstation (Syntegra, Philips, Milpitas, CA). SUV was defined as tissue concentration of FDG (kBq/mL) divided by the injected activity per weight (MBq/kg). All images were analyzed by 2 observers (EI, EE) blinded to MRI analysis. For each FDG-avid HCC nodule, the following parameters were collected: location of the tumour according to Couinaud liver segmentation, maximum SUV of the tumour (SUV_T_) and maximum SUV of the normal liver parenchyma (SUV_L_). ROIs were drawn on 5 contiguous slices around each focus of tumour uptake in order to capture the maximum SUV; in the normal liver parenchyma, ROIs were drawn at mid height, making sure that VOI outlines were restricted to areas of physiologic uptake and avoiding neighbouring sites of disease. The tumour-to-liver ratio (SUV_T/L_) was calculated for each lesion; when no clear focus of uptake was identified, SUV_T_ was considered equal to SUV_L_, and SUV_T/L_ was set at 1.

### Liver transplantation

Of the 28 patients, 24 were transplanted during the study period, while 2 patients showed tumour progression and were dropped from the waiting list, 1 patient was excluded of LT owing to aortic stenosis and 1 patient declined LT after registration. The median delay between MRI and LT was 117 days (range 1 to 446 days). The median delay between PET/CT and LT was 104 days (range, 23 to 394 days). In all cases, a bilateral subcostal incision with an upper midline extension was performed. LT was performed using preservation of the inferior vein cava (IVC) and temporary porto-caval anastomosis except when IVC resection was necessary. Reconstruction was performed in the piggyback fashion with end-to-side cava-caval anastomosis on the joined stump of the three main hepatic veins. Portal, arterial and biliary reconstructions were performed using standard techniques.

### Pathology examination – Correlation with imaging findings

Explanted liver specimens were cross-sectioned and analyzed by a pathologist (JCa) blinded from imaging results. After formalin fixation, each liver slice was further pared to less than 5 mm thickness. All liver nodules, whether benign or consistent with HCC lesions, were macroscopically located according to Couinaud classification. The largest macroscopic diameter of each HCC lesion was recorded. For all HCC, at least a complete slice of the tumour including both the centre and the periphery was sampled for microscopic analysis. After hematoxylin-eosin-saffran staining, each nodule was analyzed for the presence of tumour cells. Microscopic evaluation of all tumours included the assessment of the following: tumour differentiation according to Edmondson-Steiner classification, presence of a capsule, quantification of necrosis, presence of MVI, and presence of satellite nodules. Patients with aggressive HCC were defined as presenting either an undifferentiated HCC (Edmondson-Steiner grades 3 and 4), and/or a micro vascular invasion, or beyond Milan criteria on the explanted specimen. Then, radiologists, nuclear medicine physicians and pathologist ensured adequate correlation between imaging and pathological data in a consensus meeting. Co-localization of tumours on pathology and imaging was not only based on segmental classification but also on the identification of specific landmarks such as hepatic veins, portal branches and central or peripheral location. Only HCC nodules retrieved simultaneously on pathology and on MRI were considered for statistical analyses.

### Statistics

The correlation between DWI-MRI and PET/CT variables (ADC_T_, ADC_L_, ADC_T/L_, T1SI relative to muscle, T2SI relative to spleen, SUV_T,_ SUV_L,_ SUV_T/L_) was analyzed using Pearson’s correlation coefficients based on pair wise complete data (37 nodules in 28 patients). In addition, because of possible erroneous ADC measures within the left lobe, induced by cardiac motion, this correlation analysis was repeated only for right lobe lesions. The association between imaging features and pathological outcomes (32 nodules in 24 LT patients) were analyzed using the following methods: 1) dichotomous outcomes (within/beyond Milan criteria, absence/presence of MVI, differentiated/undifferentiated HCC) vs. dichotomous FDG uptake (PET/CT was considered positive if the ratio SUV_T/L_ was > 1.15 [16]) were analyzed based on categorical analyses using Fisher exact test; 2) dichotomous outcomes vs. continuous imaging features (DWI-MRI and PET/CT variables) were analyzed based on univariate logistic regression; 3) continuous outcomes (differentiation status) vs. all imaging features were analyzed based on univariate linear regression. A *P*-value < 0.05 was considered statistically significant. All analyses were conducted using R statistics software package.

## Results

### DWI-MRI analysis

All 37 HCC nodules were > 10 mm on MRI with a mean tumour size of 24 ± 10 mm (range, 11 to 60 mm), and the mean ROI size for ADC measurements was 5 ± 19 voxels. The mean ADC_T_ value of these lesions was 1.39 × 10^−3^ ± 0.35 × 10^−3^ mm^2^/s (range, 0.41 to 1.89 × 10^−3^ mm^2^/s). The mean ADC_L_ value was 1.36 × 10^−3^ ± 0.37 × 10^−3^ mm^2^/s (range, 0.25 to 2.33 × 10^−3^ mm^2^/s). The mean ADC_T/L_ ratio was 1.13 ± 0.63 (range, 0.32 to 4.44).

### PET/CT analysis

**Fig. 1:**
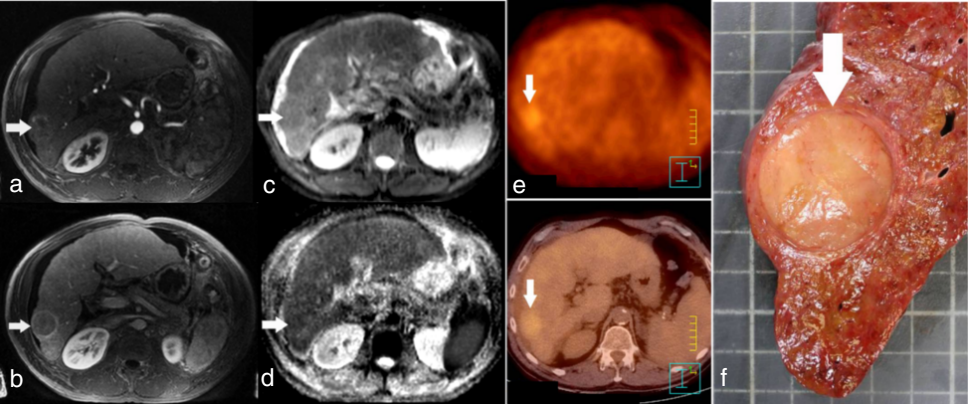
Poorly differentiated HCC in a patient with alcoholic cirrhosis, showing restricted ADC on DWI-MRI and increased uptake on the FDG-PET/CT. Transverse dynamic contrast-enhanced initial post contrast MR image shows a 30 mm large nodular enhancing lesion in the right liver on 3D GE arterial phase imaging (**a**, *arrow*) with wash-out on portal-venous phase (**b**, *arrow*). The lesion is mildly hyper intense on transverse DW image (b = 800 s/mm^2^; **c**, *arrow*) and has a low ADC (1.06 × 10^−3^ mm^2^/s) on the ADC map (**d**, *arrow*). FDG-PET/CT shows an increased tumour uptake with a SUV_T_ = 4.1 and SUV_T/L_ = 1.24 (**e**, *arrow*). The explanted liver showed an Edmondson-Steiner grade 4 HCC (**f**, *arrow*)

The mean SUV_T_ of the 37 HCC lesions retrieved on MRI was 2.9 ± 0.7 (range, 2.3 to 6.3). The mean SUV_L_ was 2.7 ± 0.3 (range, 2.3 to 3.6). The mean SUV_T/L_ was 1.08 ± 0.28 (range, 1.00 to 2.63). Overall, using the SUV_T/L_ cut-off value of 1.15, PET/CT was considered positive in 7 HCC in 7 patients: the mean SUV_T_ was 3.8 ± 1.2 in these nodules, accounting for a mean SUV_T/L_ ratio of 1.44 ± 0.53 (range, 1.19 to 2.63, Fig. 1).

### Correlation between DWI-MRI and PET/CT

**Fig. 2:**
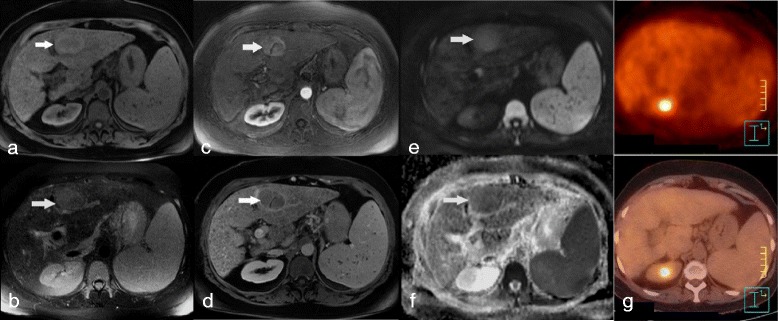
Moderately differentiated (grade 2) HCC in a patient with hepatitis B-induced cirrhosis, showing restricted ADC on DWI-MRI and no significant uptake on FDG-PET/CT. MR imaging showed a left lobe HCC with high T1 (**a**, *arrow*), high T2 (**b**, *arrow*) signal intensity, with arterial enhancement (**c**, *arrow*) followed by washout (**d**, *arrow*). The HCC showed high signal intensity on DWI image (b = 800 s/mm^2^; **e**, *arrow*) consistent with low ADC (1.26 × 10^−3^ mm^2^/s) on the ADC map (**f**, *arrow*). The lesion showed no significant FDG uptake on FDG-PET/CT (**g**)

**Fig. 3:**
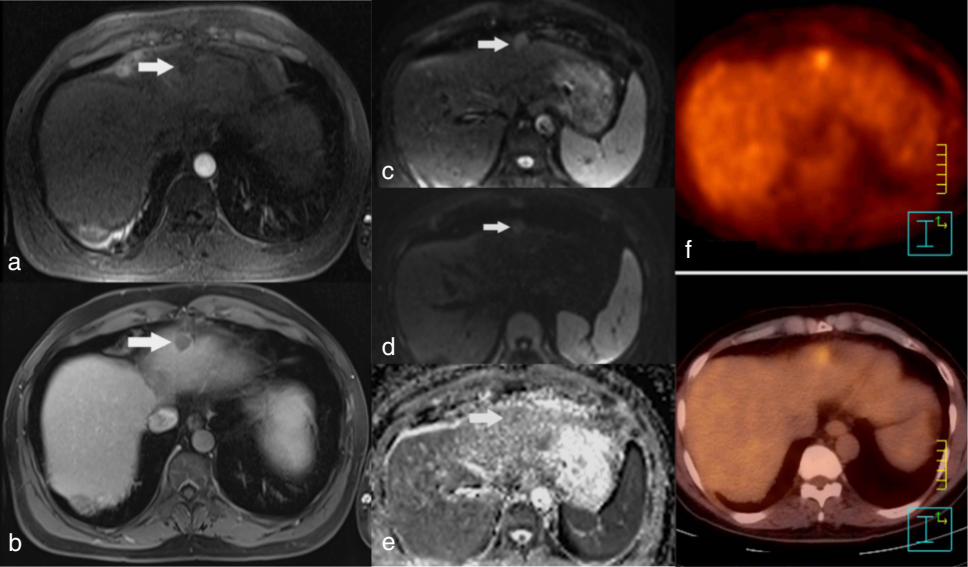
Poorly differentiated HCC in a patient with left lobe HCC, showing no restricted ADC on DWI-MRI and increased uptake on FDG-PET/CT. Dynamic 3D gradient echo T1 WI shows a non enhancing nodule on arterial phase (**a**, *arrow*), remaining hypo intense on portal phase (**b**, *arrow*) but associated to a thin peripheral enhanced rim. The nodule shows high signal intensity on DWI image with b = 0 s/mm^2^ (**c**, *arrow*) decreasing on DWI b = 800 s/mm^2^ images (**d**, *arrow*), although its signal intensity remained higher than the liver. The tumour ADC was 1.38 × 10^−3^ mm^2^/s (**e**, *arrow*), similar to that of the adjacent liver. The lesion proved positive on FDG-PET/CT (**f**)

ADC_T_ was not correlated with either SUV_T_, SUV_L_, or SUV_T/L_ ratio (Table 2, Figs. 2 and 3). ADC_L_ did not correlate either with SUV_L_, SUV_T_, or SUV_T/L_ ratio. ADC_T/L_ ratio did not correlate with either SUV_T/L_ ratio, SUV_T_, or SUV_L_. Because ADC measurements may be less reliable in the left lobe of the liver due to cardiac motion, we considered in a second analysis only lesions located within the right lobe (*n* = 28): ADC_T_ was not correlated with either SUV_T_ (*R* = 0.25, *P* = 0.3) or SUV_T/L_ ratio (*R* = 0.29, *P* = 0.21). ADC_L_ did not correlate either with SUV_T_ (*R* = 0.24, *P* = 0.4) or SUV_T/L_ ratio (*R* = 0.15, *P* = 0.57). ADC_T/L_ ratio did not correlate with either SUV_T_ (*R* = −0.18, *P* = 0.46) or SUV_T/L_ ratio (*R* = −0.12, *P* = 0.6).

**Table 2:** Correlations between ADC and SUV values. Pearson’s correlation coefficients (R) and *P*-values (P) do not show any correlation between pair wise parameters

	SUV_L_		SUV_T_		SUV_T/L_	
	R	P	R	P	R	P
ADC_L_	0. 17	0.36	0.14	0.44	0.04	0.81
ADC_T_	0.03	0.87	−0.01	0.98	−0.09	0.58
ADC_T/L_	−0.09	0.61	−0.12	0.5	−0.1	0.58

### Pathology

Gross examination revealed 32 HCC nodules in the 24 LT patients, accounting for a mean number of 1.3 lesions per patient (17 patients had 1 nodule, 6 had 2 nodules, and 1 had 3 nodules). The mean size of these HCC nodules was 21 ± 11 mm (range, 4 to 47 mm). Overall, 11 patients (46 %) met the Milan criteria based on the pathological analysis of the explanted specimens. Eight patients (33 %) showed at least one HCC lesion with MVI. The Edmondson-Steiner histological grade for LT was as follows: 2 lesions were grade 1, 7 lesions were grade 2, 17 lesions were grade 3, and 2 lesions were grade 4; four lesions could not be graded because of significant tumour necrosis.

### Correlation between imaging variables and criteria of aggressiveness

In the 28 patients on the waiting list, the median AFP level for all Child Pugh stages was 4.7 ng/mL (range, 0.7 to 13,469.0 ng/mL). The median AFP levels did not differ between Child-Pugh stages A, B and C: 5.0 ng/mL (range, 0.7 to 283.0 ng/mL), 4.6 ng/ml (range, 2.0 to 13,469.0 ng/mL), and 13.0 ng/mL (range, 2.2 to 33.0 ng/mL), respectively (*P* = NS). SUV_T/L_ ratio significantly correlated with the AFP level (*R* = 0.95, *P* < 0.0001) whereas no apparent correlation was detected between AFP and ADC_T_ or ADC_T/L_ ratio (*P* = 0.08 and *P* = 0.73, respectively). The mean AFP level was significantly higher in patients with SUV_T/L_ ratio > 1.15 than in PET/CT-negative patients (1951.9 ± 5078.9 ng/mL vs. 26.3 ± 64.0 ng/mL, respectively; *P* < 0.0001). In the 24 transplanted patients, the mean HCC tumour size was also significantly larger in patients with SUV_T/L_ ratio > 1.15 than in PET/CT-negative patients (32 ± 14 mm vs. 23 ± 7 mm; *P* < 0.0001). Tumour size did not correlate with ADC_T_ values (*P* = 0.25). In addition, positive PET/CT with SUV_T/L_ ratio > 1.15 was associated with poor differentiation on pathology (*P* = 0.04), but not with MVI or Milan criteria (*P* = NS). None of the MRI variables - including ADC_T_, ADC_T/L_, T1SI relative to muscle, and T2SI relative to spleen - correlated with pathological outcomes, including differentiation grade, MVI and Milan criteria.

## Discussion

DWI-MRI and FDG-PET/CT provide distinct functional and morphological information. To date, MRI or enhanced CT are essential for HCC lesion detection and characterization according to practice recommendations, while FDG-PET/CT is not. This study sought to evaluate the correlation between metabolic and DWI parameters, and to compare these parameters with established biomarkers of tumour aggressiveness and prognostic assessment. Increased FDG uptake within HCC nodules was associated with larger and less differentiated tumours, whereas ADC values observed in this study population appear not to correlate with AFP, differentiation on pathology and Milan criteria.

Duvoux et al. have suggested that AFP levels could predict tumour recurrence and correlated with vascular invasion and differentiation with a significant increase in 5-year risk of recurrence (50.6 %). Among patients within the Milan criteria, a subgroup of patients with AFP levels > 1000 ng/mL showed at high risk of recurrence [9]. Our study confirms that PET/CT findings correlate with AFP levels. PET/CT was considered positive if the SUV_T/L_ ratio was > 1.15, which occurred in 7 patients on the waiting list (25 %), of whom 2 demonstrated tumour progression and did not undergo LT. Higher-grade and less-differentiated tumours (according to Edmondson-Steiner classification) were associated with higher levels of FDG accumulation. Decreased levels of glucose-6-phosphatase observed in poorly differentiated HCC lead to increased FDG accumulation [10, 22]. Our results confirm the performance of FDG-PET/CT in predicting tumour aggressiveness using the explanted specimen as the reference standard. In addition, PET/CT performed once as a screening tool even as far as a median 104 days prior to LT still correlates with pathological features of HCC.

The ADC value, measured by DWI, usually reflects tumour cellularity as well as interstitial tissue assessment. The mean ADC_T_ values observed in this study are comparable to those reported in the literature [18, 23]. Similarly, ADC_T/L_ ratios observed in this study are not high, reflecting the low contrast between the tumour lesions and adjacent liver on ADC maps [24–26]. This could be partly explained by the restricted diffusion observed in liver fibrosis [27]. There have been conflicting reports regarding the link between ADC values and HCC differentiation: for some, although differences are not significant in terms of parametric ADC values, a higher signal intensity of HCC lesions in DWI is usually associated with higher pathological grades [18, 19]. However, the reasons accounting for an increase in mean ADC values vary: tumour necrosis is responsible for reduced cellularity and increased ADC [28]; on the opposite, increased necrosis is also observed in highly aggressive HCC lesions with active portions of the tumour mostly located at the periphery of the lesions [29]. Last, tumour vascularization impacts ADC measurements, especially when low b-values are included, such as in this study where 4 b-values were used, including b = 0 s/mm^2^. As multiple b-value acquisitions were not studied, the specific influence of the perfusion component of diffusion in predicting tumour aggressiveness could not be tested here. In addition, the ADC measurements encompassed the entire HCC lesions, reflecting all components of the tumour observed on a single slice rather than on the entire tumour volume, which could also have limited the performances of ADC measurements. FDG uptake on the other hand correlates with the metabolic activity of viable tumour cells. Although necrosis decreases the overall tumour uptake, the SUV is measured from the area of highest uptake within the lesion set; the SUV only considers the most aggressive tumour components. Comparing mean ADC to maximum SUV does not reflect a true comparison of the most active component of each lesion. Moreover, a majority of HCC lesions observed in our study showed similar SUV values as compared to the adjacent liver, thus reducing the dispersion of SUV values as compared to ADC measurements. The use of novel tracers such as ^18^F-fluoromethylcholine (FCH) could allow a better distribution of uptake values in HCC lesions even in well-differentiated HCC lesions.

In contrast with previous publications, this study focused on patients on waiting list for LT, using pathology of explanted specimens as the reference standard in a large majority of them. Moreover, there have been conflicting reports on the correlation between DWI and PET/CT in HCC, as tissue cellularity and glucose metabolism may represent 2 different facets of tumour biology [30]: some studies have suggested a positive correlation between tumour SUVs and ADC values [31–37]. Other authors have reported an inverse relationship between these parameters, especially when discriminating malignant from benign lesion, or when predicting the aggressiveness of some tumours [31, 33–39]. Hypercellular areas could increase impedance to water diffusion, resulting in low ADC values and high FDG uptake, while lower cellularity areas could show increased ADC with decreased FDG uptake. These relationships were reported mainly in extra-digestive tumours possibly because the influence of motion on ADC measurements is more limited in this setting. This further suggests the need to test DWI-MRI together with multitracer PET (FDG and/or FCH) in dedicated PET/MR platforms allowing simultaneous acquisitions and limited influence of motion on parametric imaging analysis [40].

Several limitations of the presented study have to be mentioned. First, the patient population was small in this monocentric retrospective study. There is currently no national recommendation for systematic FDG-PET/CT in routine before LT. Second, only patients on LT waiting list were considered eligible, which prevented inclusion of more aggressive and larger tumours. The study design further excluded patients who were treated with chemoembolization, radiofrequency, and chemotherapy before DWI-MRI because these treatments alter ADC measures [41, 42]. Interestingly, 15 patients were treated between MRI and LT, which could possibly have impacted the correlation between imaging and pathology [41, 42]. However, this did not affect the SUV and ADC correlation as all correlations were made on imaging examinations performed prior to any treatment. There was no image fusion between PET/CT and DWI-MRI; however, to our knowledge and to-date, no software enables the correct simultaneous fusion between b-maps, ADC maps and PET/CT images.

PET/CT was obtained without contrast. However this did not impact the co-registration of MRI images: all MRI lesions were detected on both FDG-PET/CT and on pathology. Additionally, it is important to note that only patients meeting the EASL-AASLD criteria were included, limiting the analysis to lesions larger than 10 mm with early enhancement on arterial phase. However, the aim of this study was not to assess the performance of each modality for the detection of HCC in patients on waiting list for LT, for which MRI plays a key role, but to study the possible predictive nature of FDG-PET/CT or DWI sequences on tumour aggressiveness. The median delay between FDG-PET/CT and MRI-DWI was 3 days with a maximum of 245 days. FDG-PET/CT and DWI-MRI datasets were evaluated separately without knowledge of the findings of the other imaging modality and without knowledge of histopathology findings.

## Conclusions

Although mean ADC and FDG-PET/CT maximum SUV of HCC in patients on waiting list for LT are not correlated, both tumour size, poor differentiation and AFP levels correlate with SUV_T/L_, suggesting that FDG-PET/CT can predict tumour aggressiveness and should probably be integrated as a covariable in a predictive outcome model of patients with HCC candidate to LT, while MRI must be used for lesion detection and characterization.

## References

[1] Allen J, Venook A (2004). Hepatocellular carcinoma: epidemic and treatment. Curr Oncol Rep..

[2] Mazzaferro V, Regalia E, Doci R, Andreola S, Pulvirenti A, Bozzetti F (1996). Liver transplantation for the treatment of small hepatocellular carcinomas in patients with cirrhosis. New Engl J Med..

[3] Klintmalm GB (1998). Liver transplantation for hepatocellular carcinoma: a registry report of the impact of tumor characteristics on outcome. Ann Surg..

[4] Jonas S, Bechstein WO, Steinmüller T, Herrmann M, Radke C, Berg T (2001). Vascular invasion and histopathologic grading determine outcome after liver transplantation for hepatocellular carcinoma in cirrhosis. Hepatology..

[5] Molmenti EP, Klintmalm GB (2002). Liver transplantation in association with hepatocellular carcinoma: an update of the International Tumor Registry. Liver Transplant..

[6] Marsh JW, Finkelstein SD, Demetris AJ, Swalsky PA, Sasatomi E, Bandos A (2003). Genotyping of hepatocellular carcinoma in liver transplant recipients adds predictive power for determining recurrence-free survival. Liver Transplant..

[7] Han K, Tzimas GN, Barkun JS, Metrakos P, Tchervenkov JL, Hilzenrat N (2007). Preoperative alpha-fetoprotein slope is predictive of hepatocellular carcinoma recurrence after liver transplantation. Can J Gastroenterol Hepatol..

[8] Kim H, Park M-S, Choi JY, Park YN, Kim M-J, Kim KS (2009). Can microvessel invasion of hepatocellular carcinoma be predicted by pre-operative MRI?. Eur Radiol..

[9] Duvoux C, Roudot-Thoraval F, Decaens T, Pessione F, Badran H, Piardi T (2012). Liver transplantation for hepatocellular carcinoma: a model including α-fetoprotein improves the performance of Milan criteria. Gastroenterology.

[10] Torizuka T, Tamaki N, Inokuma T, Magata Y, Sasayama S, Yonekura Y (1995). In vivo assessment of glucose metabolism in hepatocellular carcinoma with FDG-PET. J Nucl Med..

[11] Lam MGEH, Kwee TC, Basu S, Alavi A (2013). Underestimated role of ^18^F-FDG PET for HCC evaluation and promise of ^18^F-FDG PET/MR imaging in this setting. J Nucl Med..

[12] Cheung TT, Ho CL, Chen S, Chan SC, Poon RTP, Fan ST (2013). Reply: Underestimated role of ^18^F-FDG PET for HCC evaluation and promise of ^18^F-FDG PET/MR imaging in this setting. J Nucl Med..

[13] Pant V, Sen IB, Soin AS (2013). Role of ^18^F-FDG PET CT as an independent prognostic indicator in patients with hepatocellular carcinoma. Nucl Med Commun..

[14] Song J-Y, Lee YN, Kim YS, Kim SG, Jin SJ, Park JM (2015). Predictability of preoperative ^18^F-FDG PET for histopathological differentiation and early recurrence of primary malignant intrahepatic tumors. Nucl Med Commun..

[15] Wang S-B, Wu H-B, Wang Q-S, Zhou W-L, Tian Y, Li H-S (2015). Combined early dynamic (18)F-FDG PET/CT and conventional whole-body (18)F-FDG PET/CT provide one-stop imaging for detecting hepatocellular carcinoma. Clin Res Hepatol Gastroenterol..

[16] Lee JW, Paeng JC, Kang KW, Kwon HW, Suh K-S, Chung J-K (2009). Prediction of tumor recurrence by ^18^F-FDG PET in liver transplantation for hepatocellular carcinoma. J Nucl Med..

[17] Kornberg A, Küpper B, Tannapfel A, Büchler P, Krause B, Witt U (2012). Patients with non-[^18^F] fludeoxyglucose-avid advanced hepatocellular carcinoma on clinical staging may achieve long-term recurrence-free survival after liver transplantation. Liver Transplant..

[18] Nasu K, Kuroki Y, Tsukamoto T, Nakajima H, Mori K, Minami M (2009). Diffusion-weighted imaging of surgically resected hepatocellular carcinoma: imaging characteristics and relationship among signal intensity, apparent diffusion coefficient, and histopathologic grade. Am J Roentgenol..

[19] Heo SH, Jeong YY, Shin SS, Kim JW, Lim HS, Lee JH (2010). Apparent diffusion coefficient value of diffusion-weighted imaging for hepatocellular carcinoma: correlation with the histologic differentiation and the expression of vascular endothelial growth factor. Korean J Radiol..

[20] Mazzaferro V, Llovet JM, Miceli R, Bhoori S, Schiavo M, Mariani L (2009). Predicting survival after liver transplantation in patients with hepatocellular carcinoma beyond the Milan criteria: a retrospective, exploratory analysis. Lancet Oncol..

[21] Bruix J, Sherman M, Diseases AAftSoL (2011). Management of hepatocellular carcinoma: an update. Hepatology.

[22] Khan MA, Combs CS, Brunt EM, Lowe VJ, Wolverson MK, Solomon H (2000). Positron emission tomography scanning in the evaluation of hepatocellular carcinoma. J Hepatol..

[23] Vivarelli M, Cucchetti A, Piscaglia F, La Barba G, Bolondi L, Cavallari A (2005). Analysis of risk factors for tumor recurrence after liver transplantation for hepatocellular carcinoma: key role of immunosuppression. Liver Transplant..

[24] Le Bihan D, Breton E, Lallemand D, Aubin ML, Vignaud J, Laval-Jeantet M (1988). Separation of diffusion and perfusion in intravoxel incoherent motion MR imaging. Radiology..

[25] Lewin M, Poujol-Robert A, Boëlle P-Y, Wendum D, Lasnier E, Viallon M (2007). Diffusion-weighted magnetic resonance imaging for the assessment of fibrosis in chronic hepatitis C. Hepatology..

[26] Koinuma M, Ohashi I, Hanafusa K, Shibuya H (2005). Apparent diffusion coefficient measurements with diffusion-weighted magnetic resonance imaging for evaluation of hepatic fibrosis. J Magn Reson Imaging..

[27] Luciani A, Vignaud A, Cavet M, Nhieu JTV, Mallat A, Ruel L (2008). Liver cirrhosis: intravoxel incoherent motion MR imaging--pilot study. Radiology..

[28] Mannelli L, Kim S, Hajdu CH, Babb JS, Clark TWI, Taouli B (2009). Assessment of tumor necrosis of hepatocellular carcinoma after chemoembolization: diffusion-weighted and contrast-enhanced MRI with histopathologic correlation of the explanted liver. Am J Roentgenol..

[29] Kirimlioglu H, Dvorchick I, Ruppert K, Finkelstein S, Marsh JW, Iwatsuki S (2001). Hepatocellular carcinomas in native livers from patients treated with orthotopic liver transplantation: biologic and therapeutic implications. Hepatology..

[30] Ahn SJ, Park M-S, Kim KA, Park JY, Kim I, Kang WJ (2013). ^18^F-FDG PET metabolic parameters and MRI perfusion and diffusion parameters in hepatocellular carcinoma: a preliminary study. PloS One..

[31] Punwani S, Taylor SA, Saad ZZ, Bainbridge A, Groves A, Daw S (2013). Diffusion-weighted MRI of lymphoma: prognostic utility and implications for PET/MRI?. Eur J Nucl Med Mol Imaging..

[32] Olsen JR, Esthappan J, DeWees T, Narra VR, Dehdashti F, Siegel BA (2013). Tumor volume and subvolume concordance between FDG-PET/CT and diffusion-weighted MRI for squamous cell carcinoma of the cervix. J Magn Reson Imaging..

[33] Gong N-J, Wong C-S, Chu Y-C, Gu J (2013). Treatment response monitoring in patients with gastrointestinal stromal tumor using diffusion-weighted imaging: preliminary results in comparison with positron emission tomography/computed tomography. NMR biomed..

[34] Regier M, Derlin T, Schwarz D, Laqmani A, Henes FO, Groth M (2012). Diffusion weighted MRI and ^18^F-FDG PET/CT in non-small cell lung cancer (NSCLC): does the apparent diffusion coefficient (ADC) correlate with tracer uptake (SUV)?. Eur J Radiol..

[35] Wong CS, Gong N, Chu Y-C, Anthony M-P, Chan Q, Lee HF (2012). Correlation of measurements from diffusion weighted MR imaging and FDG PET/CT in GIST patients: ADC versus SUV. Eur J Radiol..

[36] Park SH, Moon WK, Cho N, Chang JM, Im S-A, Park IA (2012). Comparison of diffusion-weighted MR imaging and FDG PET/CT to predict pathological complete response to neoadjuvant chemotherapy in patients with breast cancer. Eur Radiol..

[37] Wu X, Kellokumpu-Lehtinen P-L, Pertovaara H, Korkola P, Soimakallio S, Eskola H (2011). Diffusion-weighted MRI in early chemotherapy response evaluation of patients with diffuse large B-cell lymphoma--a pilot study: comparison with 2-deoxy-2-fluoro- D-glucose-positron emission tomography/computed tomography. NMR Biomed..

[38] Rahmouni A, Luciani A, Itti E. MRI and PET in monitoring response in lymphoma. Cancer Imaging. 2005;5 Spec No A:S106–12. doi:10.1102/1470-7330.2005.0038. 10.1102/1470-7330.2005.0038 PMC1665309 16361125

[39] Ho K-C, Lin G, Wang J-J, Lai C-H, Chang C-J, Yen T-C (2009). Correlation of apparent diffusion coefficients measured by 3T diffusion-weighted MRI and SUV from FDG PET/CT in primary cervical cancer. Eur J Nucl Med Mol Imaging..

[40] Catalano OA, Rosen BR, Sahani DV, Hahn PF, Guimaraes AR, Vangel MG (2013). Clinical impact of PET/MR imaging in patients with cancer undergoing same-day PET/CT: initial experience in 134 patients--a hypothesis-generating exploratory study. Radiology..

[41] Li SP, Padhani AR (2012). Tumor response assessments with diffusion and perfusion MRI. J Magn Reson Imaging..

[42] Bonekamp S, Corona-Villalobos CP, Kamel IR (2012). Oncologic applications of diffusion-weighted MRI in the body. J Magn Reson Imaging..

